# The impact of COVID-19 mobility restrictions on dengue transmission in urban areas

**DOI:** 10.1371/journal.pntd.0012644

**Published:** 2024-11-25

**Authors:** Jorge L. B. Araújo, Rafael Bomfim, Cesar I. N. Sampaio Filho, Luciano P. G. Cavalcanti, Antonio S. Lima Neto, José S. Andrade, Vasco Furtado

**Affiliations:** 1 Laboratório de Ciência de Dados e Inteligência Artificial, Universidade de Fortaleza, Fortaleza, Ceará, Brazil; 2 Departamento de Física, Universidade Federal do Ceará, Fortaleza, Ceará, Brazil; 3 Programa de Pós-Graduação em Saúde Coletiva, Universidade Federal do Ceará, Ceará, Brazil; 4 Escola de Saúde Pública do Ceará, Fortaleza, Ceará, Brazil; 5 Secretaria Executiva de Vigilância em Saúde, Secretaria da Saúde do Ceará, Fortaleza, Ceará, Brazil; 6 Empresa de Tecnologia da Informação do Ceará, Governo do Estado do Ceará, Fortaleza, Ceará, Brazil; Columbia University, UNITED STATES OF AMERICA

## Abstract

During the COVID-19 pandemic, governments have been forced to implement mobility restrictions to slow down the spread of SARS-CoV-2. These restrictions have also played a significant role in controlling the spread of other diseases, including those that do not require direct contact between individuals for transmission, such as dengue. In this study, we investigate the impact of human mobility on the dynamics of dengue transmission in a large metropolis. We compare data on the spread of the disease over a nine-year period with data from 2020 when strict mobility restrictions were in place. This comparison enables us to accurately assess how mobility restrictions have influenced the rate of dengue propagation and their potential for preventing an epidemic year. We observed a delay in the onset of the disease in some neighborhoods and a decrease in cases in the initially infected areas. Using a predictive model based on neural networks capable of estimating the potential spread of the disease in the absence of mobility restrictions for each neighborhood, we quantified the change in the number of cases associated with social distancing measures. Our findings with this model indicate a substantial reduction of approximately 72% in dengue cases in the city of Fortaleza throughout the year 2020. Additionally, using an Interrupted Time Series (ITS) model, we obtained results showing a strong correlation between the prevention of dengue and low human mobility, corresponding to a reduction of approximately 45% of cases. Despite the differences, both models point in the same direction, suggesting that urban mobility is a factor strongly associated with the pattern of dengue spread.

## Author summary

In our study, we discovered a surprising connection between mobility restrictions during the COVID-19 pandemic and the reduction in the spread of dengue, a mosquito-borne disease. We analyzed data from a large city over several years, including the year 2020, when these restrictions were rigorously in place. Our focus was to understand how the limitation of people’s movements influenced the dynamics of dengue transmission. The results were revealing. We observed that, with fewer people moving around, there was a delay in the emergence of dengue in some areas and a decrease in cases in initially affected regions. We utilized both neural network and Interrupted Time Series (ITS) models to predict potential outcomes in the absence of mobility restrictions. Our analysis with the neural network model estimated that approximately 20,000 cases of dengue were prevented, while the ITS model indicated a reduction of about 7,000 cases during this period. This study expands our understanding of dengue, highlighting the importance of human mobility patterns in the spread of this disease. These findings are especially significant for urban areas and have important implications for public health strategies in similar contexts.

## Introduction

Dengue (DENV) is a widespread arbovirus, affecting 40% of the global population in areas at risk of infection [1]. It is a systemic viral disease transmitted to humans through the bite of infected Aedes genus mosquitoes. The conferred immunity is serotype-specific and long-lasting. Given that there are four serotypes of the dengue virus (DENV1, DENV2, DENV-3, DENV-4), an individual can experience up to four infections during their lifetime. Nearly 390 million dengue cases are reported each year [2], resulting in 40,000 deaths.

Fortaleza if the fifth most populous city in Brazil with approximately 2.4 million inhabitants. Dengue emerged as a public health issue in Fortaleza in 1986, with the introduction of dengue virus serotype 1 (DENV-1). The situation escalated when dengue virus serotype 2 (DENV-2) was introduced, rapidly becoming the dominant serotype and leading to the first significant epidemic in 1994. A seroepidemiological survey conducted during this period revealed a high attack rate, with an estimated 44% of Fortaleza’s population having been infected by DENV-2 [3]. Since then, the local scenario has been characterized by the alternation of some epidemic years interspersed with years in which a classic pattern of endemicity was established. Periods of high transmission are almost always associated with the introduction or reintroduction of serotypes when there is a substantial proportion of susceptible individuals in the population [4].

In 2020, due to the COVID-19 pandemic, the world experienced unprecedented mobility restrictions aimed at reducing SARS-CoV-2 transmission. Most cities implemented some form of social contact restriction, such as prohibiting large gatherings and social meetings, reducing crowding on public transportation, and fully or partially limiting citizens’ movements within the city. The scientific literature has long explored the impact of human mobility on disease transmission [5–8]. Specifically, for SARS-CoV-2, mobility restrictions have influenced virus transmission in various locations worldwide [9–13]. In particular, Hâncean *et al*. [14] studied the impact of human mobility networks on the global spread of COVID-19, emphasizing how migration and tourism contributed to virus transmission across 203 countries. Their findings suggest that a mix of mobility and geographic factors played a significant role in the global transmission dynamics, which has implications for public health interventions and the management of human circulation. Lockdowns have also affected the transmission of other diseases spread through direct contact, such as influenza [15]. However, assessing the impact of mobility on dengue, which is transmitted through mosquitoes, is more complex. Three recent studies have presented conflicting conclusions. Cavani *et al*. [16] adopted an agent-based model to examine the evolution of dengue cases during the 2020 lockdown, utilizing data on mobility and dengue incidence in Iquitos, Peru. The authors argue that the lockdown may have increased dengue cases due to the higher transmission rate of the virus in residential environments. In contrast, Liyanage *et al*. [17] examining dengue cases in Sri Lanka throughout 2020 concluded that the lockdown led to a decrease in cases, which directly correlated with reduced mobility, as evidenced by Google mobility data [18]. Broadening the discussion, the study on dengue in Caldas, Colombia, presents an intriguing contrast to previous findings. While the last two papers report divergent outcomes regarding the impact of lockdowns, Carolina *et al*. [19] reveal an additional complexity. Using a metapopulation propagation model, the authors concluded that isolating specific endemic nodes can reduce dengue cases, but paradoxically, total isolation might increase them.

In this work, we deepen our understanding of the relationship between mobility restrictions and dengue transmission by analyzing ten years of dengue cases in Fortaleza. Our focus is on the year 2020 when mobility was partially restricted from March 20th to May 7th and completely banned from May 8th to May 31st (Lockdown). We employ a predictive model based on neural networks and an Interrupted Time Series (ITS) model to quantify the potential reduction in cases in the city using historical dengue case data and population mobility data from Fortaleza’s bus system. Our results show that the significant decrease in bus system smart-card validations correlates with a substantial reduction in dengue cases. Moreover, the spread of the disease in neighborhoods varied but displayed a noticeable pattern of reduced contagion due to mobility restrictions. This reduction was more pronounced in neighborhoods with historically higher infection rates.

In the subsequent sections, we first present the methodological resources utilized in this study, highlighting the data used and the predictive models of time series, including the neural network and the ITS models. The results section then presents our key findings, focusing on the observed reductions in dengue cases following mobility restrictions. Finally, we present our main conclusions, emphasizing the observed reduction in dengue cases due to mobility restrictions and comparing our findings with other qualitative information that shows the importance of urban mobility in the spread of dengue, as evidenced by their strong correlation revealed in this study.

## Methodological resources

### Datasets

Data regarding dengue cases were provided by the Fortaleza Health Secretariat and pertain to notifications made in hospitals and public health clinics. The records include the neighborhood in which the patient resides, as well as the notification date, allowing for data aggregation among the 119 neighborhoods of Fortaleza. The complete historical series can be accessed using the Sistema de Monitoramento Diário de Agravos—SIMDA (Daily Disease Occurrence Monitoring System) [20]. From the SIMDA data, we extracted information from 2011 to 2020, totaling 159,310 dengue cases during this period.

Data on human mobility in the city originates from validations made with the smartcard system for city buses. The geographic position of the validation is captured from GPS receivers installed on buses, which allows for estimating the origin of a given ride path using the algorithm described by Caminha *et al*. [21]. Bus mobility data strongly correlates with data captured by Google and provided in the COVID-19 Community Mobility Reports [18]. Moreover, other modes of movement also correlate with this data. Smartcard validations enable the estimation of the number of rides departing from each neighborhood during the pandemic, highlighting their importance in the spread of dengue. The recorded validations cover the period from January 1, 2020, to December 31, 2020.

Data and codes used in this paper are available in: https://github.com/rafaellpontes/dengue-code-paper).

### Predictive model: Neural network

Predictions involving time series of dengue cases have been previously explored with various other machine learning models, as discussed in the work of Bomfim *et al*. [22]. It was shown in this study that neural networks can achieve good performance for predicting time series of dengue cases. In this context, we employ a specialized neural network model to address the prediction of dengue cases across different neighborhoods.

**Fig 1:**
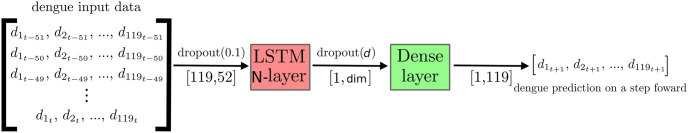
Prediction models based on neural networks. Neural network architecture illustration. The architecture has as input a matrix of size [119, 52] containing the time series of weekly dengue cases of size 52 for each of the 119 neighborhoods. The temporal and spatial dependence between these values is learned in the LSTM layer with *dim* neurons and the prediction for the 119 neighborhoods one-step-ahead is made in the Dense layer with 119 neurons. A dropout of *d*% is applied before each *N* LSTM and Dense layers to prevent over-fitting. *Dim* represents the number of neurons, *d* the dropout value, and *N* the number of LSTM layers. These values were selected via Grid Search during the training process. The range of values used in Grid Search to define the neural network’s hyperparameters is presented in S1 Table.

To predict dengue cases for the 119 neighborhoods, the model was trained using dengue case data that was divided into test, validation, and training sets according to Table 1. The neural network hyper-parameters were adjusted using the Grid Search method [23], a method that performs a full scan of all predefined possibilities of parameters and finds the best combination (e.g., Long Short-Term Memory (LSTM) layer dimensions, batch size, dropout values). The neural network architecture, shown in Fig 1, takes as input the time series of the number of dengue cases per week for a 52-week history (annual cycle) for all 119 neighborhoods.

**Table 1:** Data Division: The data was split into test, evaluation, and training data. The test data is used for prediction and analysis purposes, the evaluation data for adjusting the model parameters during the training phase, and the training data to learn the historical pattern of dengue case evolution. For consistency, we chose one epidemic year (e.g., 2016) and one non-epidemic year (e.g., 2018) for the evaluation phase, ensuring the model was tested across different outbreak scenarios.

Test	Evaluation	Training
2011	2016, 2018	2012,2013,2014,2015,2017,2019
2012	2016, 2018	2011,2013,2014,2015,2017,2019
2013	2016, 2018	2011,2012,2014,2015,2017,2019
2014	2016, 2018	2011,2012,2013,2015,2017,2019
2015	2016, 2018	2011,2012,2013,2014,2017,2019
2016	2015, 2018	2011,2012,2013,2014,2017,2019
2017	2016, 2018	2011,2012,2013,2014,2015,2019
2018	2016, 2019	2011,2012,2013,2014,2015,2017
2019	2016, 2018	2011,2012,2013,2014,2015,2017
2020	2016, 2018	2011,2012,2013,2014,2015,2017,2019

The temporal and spatial dependence between the evolution of dengue cases is learned in the LSTM layer (or stacked LSTM) with a dimension of 150, where the dimension represents the number of neurons in this layer of the neural network. The output of the LSTM consists of values that serve as input for a Dense Layer with a dimension of 119, which predicts dengue cases for all 119 neighborhoods for the following week. The prediction is performed recursively, meaning that the prediction of dengue cases for one week serves as input to the model for predicting the following week. The list of all parameters adjusted by the Grid Search and the predefined values for each are available in the Supporting Information (S1 Table).

### Predictive model: Interrupted Time Series

In order to corroborate the previous results obtained with neural networks and provide additional evidence for the strong correlation between the reduced numbers of dengue cases and the decrease in mobility during the COVID-19 pandemic, we also performed an analysis with an Interrupted Time Series (ITS) model [24] on the data of dengue cases for the years 2019 and 2020. The ITS analysis is a powerful quasi-experimental design used to evaluate the impact of an intervention by investigating changes in the trend of a time series before and after the intervention. This method is particularly valuable in scenarios where randomized controlled trials are not feasible.

ITS involves collecting data at multiple time points before, during, and after the intervention, allowing for the assessment of both immediate and long-term effects. The analysis follows two steps. First, by means of a segmented regression model that includes a variable to quantify the effect of the intervention, an estimate of the evolution of dengue cases is obtained for the entire period of analysis. If this inference is statistically validated, another regression is performed to estimate the number of dengue cases disregarding the occurrence of the intervention. This so-called “counterfactual estimation” is then compared with the observed time series to account for the difference in the number of cases during both the intervention and post-intervention periods.

As in Ref. [17], we combined ITS with Generalized Additive Models (GAMs), a flexible class of regression models that allow for non-linear relationships between dependent and independent variables by incorporating smooth functions of the predictors. These models extend generalized linear models (GLMs) by replacing the linear predictor with a sum of smooth functions, providing a powerful tool for capturing complex patterns in the data. GAMs are particularly useful when the relationship between predictors and the response variable is not strictly linear, allowing for more accurate modeling of real-world phenomena. Furthermore, it is important to note that the results from both models adopted here, LSTM and ITS, were obtained without the input of mobility data. Instead, they relied on historical transmission trends from years without mobility restrictions. As a result, the models capture the natural dynamics of dengue transmission under typical urban mobility conditions. Future work should explore integrating mobility data to provide more nuanced predictions.

## Results

**Fig 2:**
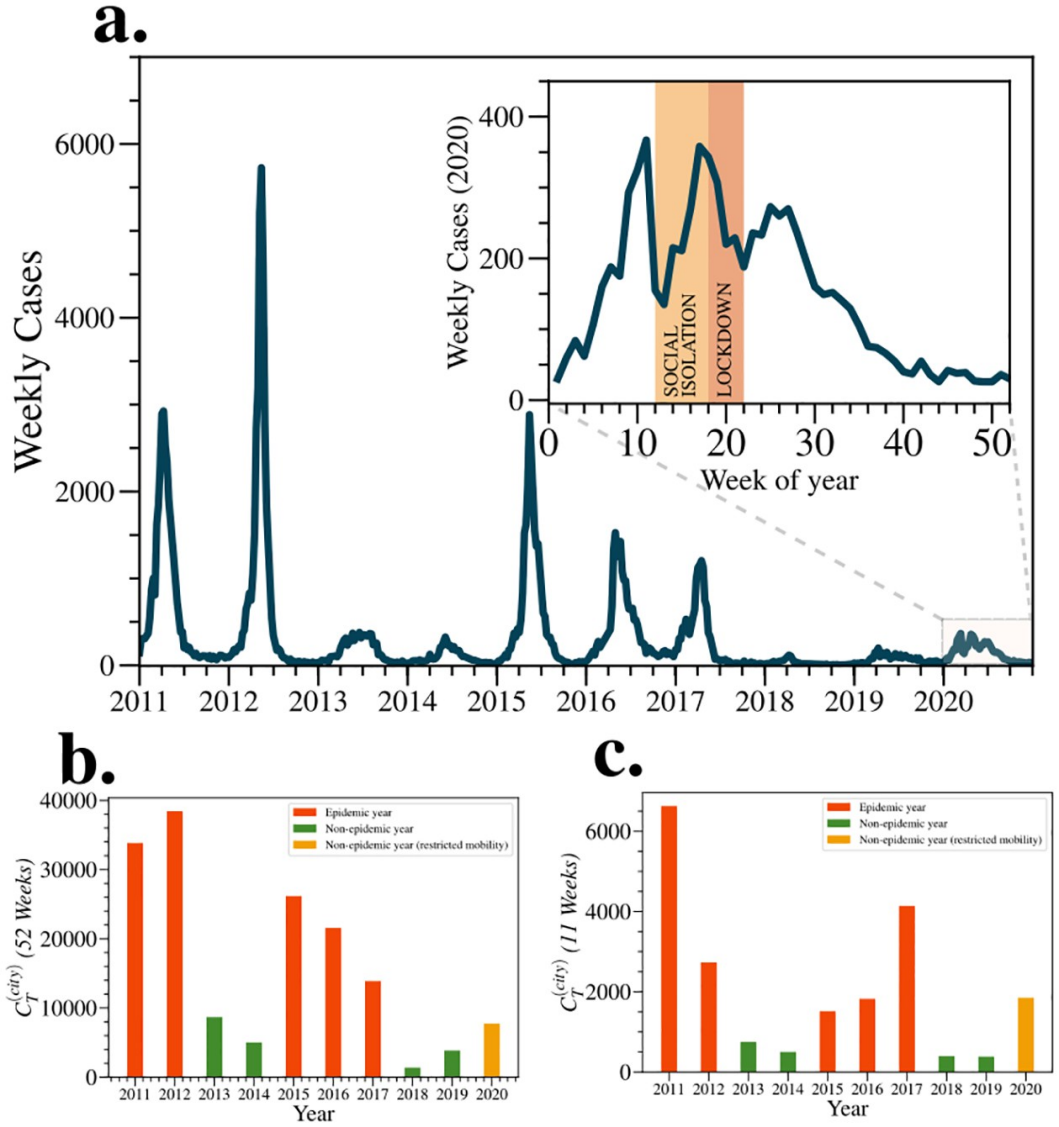
Time series of the weekly dengue cases observed in the city of Fortaleza during the interval [2011, 2020]. (a) Typically, the annual behavior of dengue epidemic curves is well represented by bell-shaped functions, where the period of highest incidence is usually observed between April and June (Week 9 to Week 20). However, in 2020, urban mobility was dramatically reduced by governmental actions imposing restrictions on mobility to mitigate COVID-19 dissemination. Restrictions such as Social Isolation (03–20-2020 to 05–07-2020) and Lockdown (05–08-2020 to 05–31-2020) have substantially influenced the shape of the curve of cases, causing different moments of highest contamination (Week 9 and Week 16). Orange time zones between Weeks 12 and 24 of 2020 represent the mobility restriction periods. In (b), we show the total number of cases $C_{T}^{\left(city\right)}$ observed in the city of Fortaleza at different moments of contagion. The years 2011, 2012, 2015, 2016, and 2017 are dubbed epidemic since they exhibit a large quantity of confirmed cases. The remaining years are considered non-epidemic. To draw a comparison with the timeframe immediately preceding the implementation of mobility restrictions in 2020, we limit our analysis in (c) to the total case count for the initial 11 weeks of dengue infection each year. Although 2020 initially demonstrates a contamination pattern akin to epidemic years, suggesting that high rates of serotype DENV2 contagion would occur in the subsequent weeks of the year, the ultimate figures do not validate that projection. Consequently, by year-end, 2020 is categorized as a non-epidemic year.

Fig 2a shows the time series of weekly dengue cases over a 10-year period. Typically, the number of cases increases in early March, coinciding with the rainy season. The subsequent three to four months are often associated with rapid mosquito proliferation, leading to high transmission rates. After this period, infestation tends to decrease. The time evolution curve of dengue cases is in a bell-shaped form, where there is a peak of maximum infection throughout the year of infestation. As show in the inset of Fig 2a, however, the pattern of dengue cases in 2020 demonstrated a distinct profile, revealing a split peak in its evolution. The first peak occurred after the end of February, and the second one took place during the lockdown. Within the city’s boundaries, the distribution of dengue cases reveals marked heterogeneity, reflecting diverse patterns of transmission across different neighborhoods. Based on our analysis, we observe a particularly interesting trend: the neighborhoods consistently ranked highest in dengue case numbers remain largely unchanged over the years. In essence, those neighborhoods that are recurrently the most affected by dengue consistently occupy the top ranks. This underscores a sustained and robust transmission intensity over time. Complementary analysis available in the Supporting Information includes a series of annual maps illustrating the distribution of dengue cases by neighborhood (S1 Fig), as well as a correlogram that quantifies the stability in neighborhood ranking through Spearman’s correlation (S2 Fig), reinforcing the persistence of transmission patterns over time.

Fig 2b shows the total number of cases for each year, in the city, $C_{T}^{\left(city\right)}$. Years 2011, 2012, 2015, 2016, and 2017 are considered epidemic, with case numbers ranging between 13,000 and 37,000, while years 2013, 2014, 2018, and 2019 are characterized as non-epidemic due to the low number of cases. For example, 2018 had only 1,385 cases.

Variations in the incidence of dengue cases within Fortaleza can be attributed to the fluctuating susceptibility of the population to different serotypes of the dengue virus based on empirical evidence. Historically, all four known dengue serotypes have been identified in the city. The 2011 epidemic primarily stemmed form the reintroduction of dengue virus serotype 1 (DENV-1), while the 2012 epidemic was predominantly driven by the introduction of serotype 4 (DENV-4). In 2020, there was a resurgence of serotype 2 (DENV-2), previously the dominant serotype in 2008, causing around 35,000 cases. Initial data in 2020 suggested a pattern similar to previous epidemics, indicating the potential for a significant outbreak. Fig 2c displays the case numbers from 2011 to 2020 for the first eleven weeks of each year, showing higher numbers in 2020 compared to 2015. Contrary to expectations, however, the disease’s spread in 2020 slowed, resulting in only 7,753 cases by year-end. This unexpected development is hypothesized to be primarily due to mobility restrictions, which potentially prevented 2020 from escalating into a full-blown epidemic. This hypothesis is further supported by an analysis of bus smart-card validation data, which is discussed in the subsequent sections.

**Fig 3:**
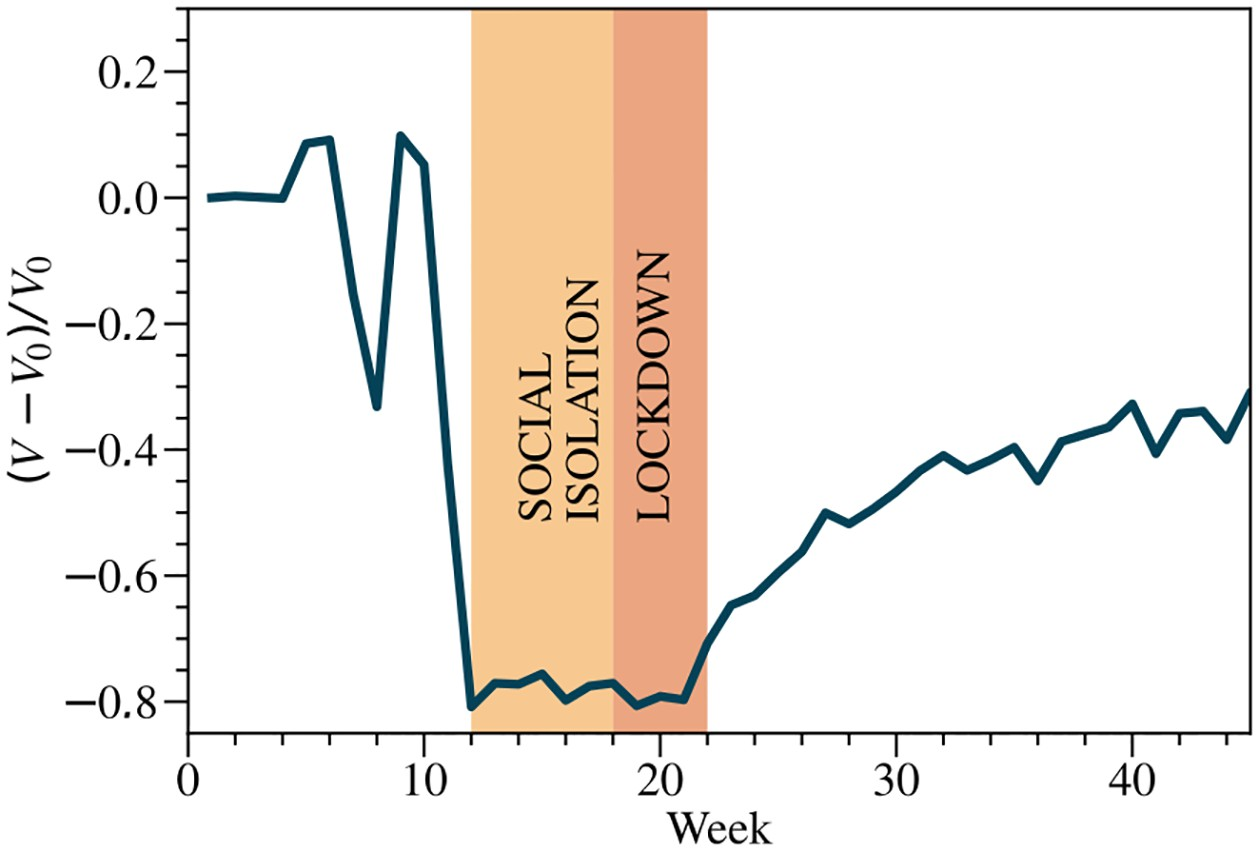
Time series of validations on buses using *smart-cards* during the COVID-19 pandemic period in 2020. The solid curve corresponds to the ratio (*V* − *V*_0_)/*V*_0_, where *V* is the number of weekly validations and *V*_0_ is a baseline given by the time average in the period [01–03-2020, 01–13-2020]. During the pandemic period, it is possible to observe a quick decrease in urban mobility in intervals [03–20-2020, 05–07-2020] (week 12—week 18) and [05–08-2020, 05–31-2020] (week 18—week 22), corresponding to *Social Isolation* and *Lockdown*, respectively. After the lockdown, a process of partial reopening of commercial establishments took place leading to a slow return to the mobility patterns usually observed. Orange time zones within the interval [week 12 to week 22] represent the intervals of mobility restrictions.

Fig 3 shows the series of validations *V* processed during the pandemic period, compared to the characteristic value *V*_0_ (baseline) related to the total number of validations in the pre-pandemic period, between 01–05-2020 and 01–12-2020. Typically, the time evolution of urban mobility profiles resembles those observed at other geographic scales [25]. As expected, the mobility behavior remains constant before restrictions, with variations due to regional holidays also captured in these data. Upon the implementation of the first mobility restriction regime (*Social Isolation*), a dramatic change in urban mobility behavior is observed. This reduction remains practically constant until the end of the second restriction period (*Lockdown*). Social Isolation (Weeks 12–18, State Decree 33,519) refers to the period when certain non-essential services, such as schools, gyms, and universities, were closed. During this period, there was a significant reduction in the number of rides, but people were not forced to stay at home. Conversely, during the lockdown period (Weeks 18–22, under State Decree 33,574), all previous restrictions were reinforced, and people were required to stay at home. Following the periods of mobility restriction, commercial establishments gradually reopened, allowing the number of bus validations to return to a level roughly equivalent to the baseline.

### Estimation of prevented dengue cases

Initially, we present the results from the neural network model, which provided a strong basis for understanding the impact of mobility restrictions on dengue cases. Following this, we introduce the analysis using the Interrupted Time Series (ITS) model to corroborate and expand upon the findings from the neural network analysis.

In order to investigate the impact of mobility restrictions on the transmission of dengue, we employ a predictive model based on neural networks, explicitly designed to handle this type of temporal data. Such a predictive model leverages a long short-term memory (LSTM) architecture [22], trained on historical dengue case data from 2011 to 2019 following the distribution of training, validation and testing from Table 1. The model is utilized to estimate the potential number of cases as if there had been no mobility restrictions in 2020. The predictions initiates from the eleventh week of 2020, made on a weekly basis at the granularity of neighborhood.

**Fig 4:**
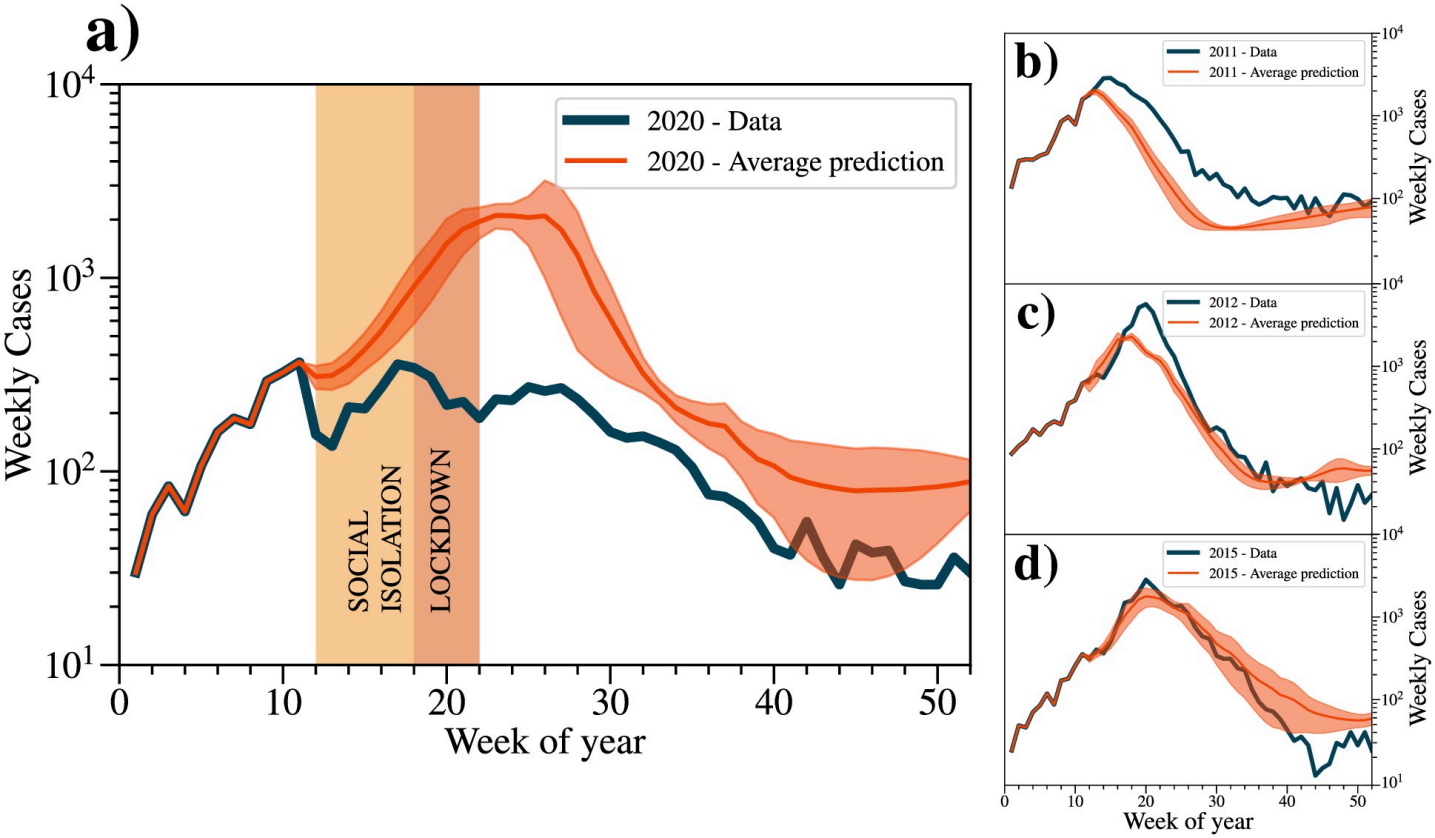
Time series of the number of dengue cases (actual and predicted) for the city of Fortaleza in the years 2020 (a) and for the epidemic years of 2011 (b), 2012 (c), and 2015 (d). The solid blue curves in the graphs represent the actual time series data, while the red curves represent the averages of the total cases for five predictions made for each year, along with the 90% confidence interval (C.I. 90%). In 2020, there were a total of 7,753 cases recorded. However, the neural network model predicted 27,792 cases, following a typical pattern observed in epidemic years.

We show in Fig 4a–4d the curves of cases predicted by the model compared to the real data measured throughout 2020 and the epidemic years 2011, 2012 and 2015, respectively (see all actual and predicted years in S3 Fig). It can be seen that the model satisfactorily captured the trend of dengue case growth in the last three cases. Notably, the weekly predictions for 2020 shown in Fig 2a follow a pattern that is similar to those of epidemic years with a consistent increase in the number of cases until the peak at week 26, and then reaches its lowest point in the middle of week 46. Remarkably, although the neural network predicted a total of 27,792 cases of dengue in 2020, the actual observed number was only $C_{T}^{\left(2020\right)}=7,753$. This incidentally provides evidence that the restrictive measures on urban mobility, initially implemented to curb COVID-19 transmission, also led to a substantial reduction of approximately 72% in dengue cases in the city of Fortaleza.

To further validate the impact of mobility restrictions on dengue cases, we applied the ITS model to the weekly dengue cases in 2019 and 2020, with the intervention period defined from March 20, 2020, to May 31, 2020. The ITS analysis involved two main steps. Initially, a segmented regression model that included a variable to measure the intervention’s impact was applied, allowing us to capture the underlying trend and seasonality of dengue cases throughout the study period. Upon statistical validation of this model, a counterfactual estimation was conducted to project the number of dengue cases without considering the intervention during the entire period after the pre-intervention phase, specifically from March 20, 2020, to December 31, 2020. This allows for a comparison between observed and predicted cases to evaluate the intervention’s effect. Specifically, a Poisson GAM with a log link function was employed:

$$\begin{array}{c} \text{log}\left(E\left(Y_{t}\right)\right)=\beta_{0}+s_{1}\left(Year\right)+s_{2}\left(Week\right)+\beta_{1}\cdot \text{Lockdown}, \end{array}$$ (1)

where *Y*_*t*_ is the number of dengue cases at time *t*, *E*(*Y*_*t*_) is the expected number of dengue cases, *β*_0_ is the intercept, and *s*_1_(*Year*) and *s*_2_(*Week*) are smooth functions capturing the effects of the year and the week, respectively, using penalized regression splines. This approach allows for flexible modeling of non-linear relationships in the data, ensuring the model can adapt to underlying trends and seasonal variations in dengue cases, providing robust predictions for the entire time series. Moreover, the Poisson distribution is appropriate for count data, such as the number of dengue cases, while the log link function ensures that the predicted values are non-negative. The *Lockdown* variable is a binary indicator that takes the value 1 during the intervention period and 0 otherwise, and *β*_1_ represents the coefficient that quantifies the impact of the intervention. By including this term, the model adjusts the expected number of dengue cases based on whether the time point falls within the intervention period, thereby isolating and quantifying the intervention’s effect on reducing dengue incidence.

**Fig 5:**
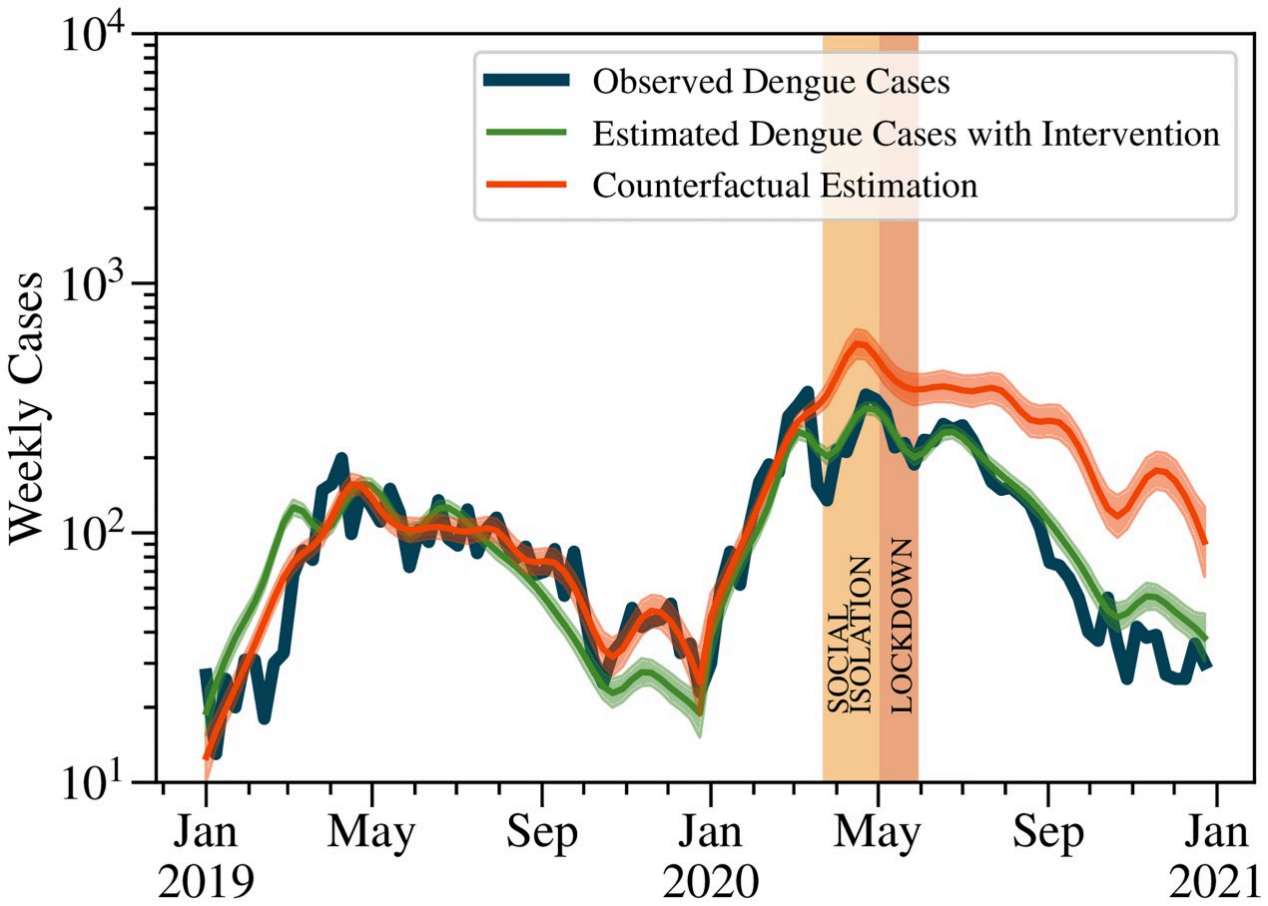
Interrupted Time Series analysis using Poisson GAM with Bootstrapped confidence intervals. This figure illustrates the observed and the predicted dengue cases intervention as well as the predicted dengue cases without intervention over time, namely, the counterfactual estimation. The solid blue curve represents the observed weekly dengue cases. The red curve represents the predicted cases assuming no intervention, while the green curve represents the predicted cases with the intervention. The shaded areas indicate the 95% confidence intervals for the predicted values. The actual number of cases in 2020 was 7,753, closely matching the predicted cases with intervention (7,752), whereas the prediction without intervention was significantly higher at 14,077 cases.

The results of the ITS analysis are shown in Fig 5. The GAM analysis indicated that all predictor terms, including the intervention, were statistically significant with p-values less than 0.001. The effective degrees of freedom for the year and week terms were 5.3 and 12.6, respectively, indicating that the model captured substantial non-linear trends and seasonal effects. The model fitting the pre-intervention data demonstrated a strong performance, explaining approximately 83% of the variability in the number of cases, as indicated by a Pseudo R-Squared of 0.8344.

The post-intervention analysis focused on evaluating the impact of the intervention on dengue cases by comparing the actual cases with predicted cases both with and without the intervention. The Poisson (GAM) was used to predict the number of dengue cases for the entire time series from January 1, 2019, to December 31, 2020, including the intervention period from March 20, 2020, to May 31, 2020. For the entire year 2020, the actual number of dengue cases was 7,753. The predicted cases with the intervention were 7,752, closely matching the actual cases, therefore indicating that the model accurately captured the intervention’s impact. In contrast, the predicted cases without the intervention were significantly higher at 14,077. This substantial difference between the predicted cases with and without the intervention clearly indicates the strong correlation between the restrictions in urban mobility and a substantial reduction of 45% in the incidence of dengue cases. The close alignment of the actual cases with the predicted cases with intervention further validates the model’s reliability in assessing the intervention’s impact. Finally, although more conservative in the reduction estimate of dengue cases, the ITS results are consistent with the predictions obtained using a neural network methodology, as both models reveal the potential severity of a dengue outbreak in 2020 in the absence of social isolation and lockdown measures. In Table 2, several key indicators comparing the observations made by the LSTM and ITS models are summarized. The table highlights important findings from each model, including the total number of predicted dengue cases, the total number of cases observed at the peak, the time at which the peak occurs, and the effective percentage of dengue cases prevented due to urban mobility restrictions.

**Table 2:** Comparison of LSTM and ITS model predictions. The table provides a detailed comparison between the predictions of the LSTM and ITS, namely the total predicted dengue cases for 2020, the predicted peak dengue cases, and the predicted time to peak. Additionally, the effective reduction in dengue cases is presented, highlighting the strong correlation with the lockdown measures. The time difference between the predicted and real peak times in the dengue case series is also compared.

Description	LSTM	ITS
Total number of predicted dengue cases	27,792	14,077
Total number of predicted dengue cases at the peak	2101.6	572.0
Time point at which the predicted dengue case series reaches its peak (week of year)	23	16
Effective reduction in dengue cases	72%	45%

### The impact of restrictions on mobility on the rate of contagion

**Fig 6:**
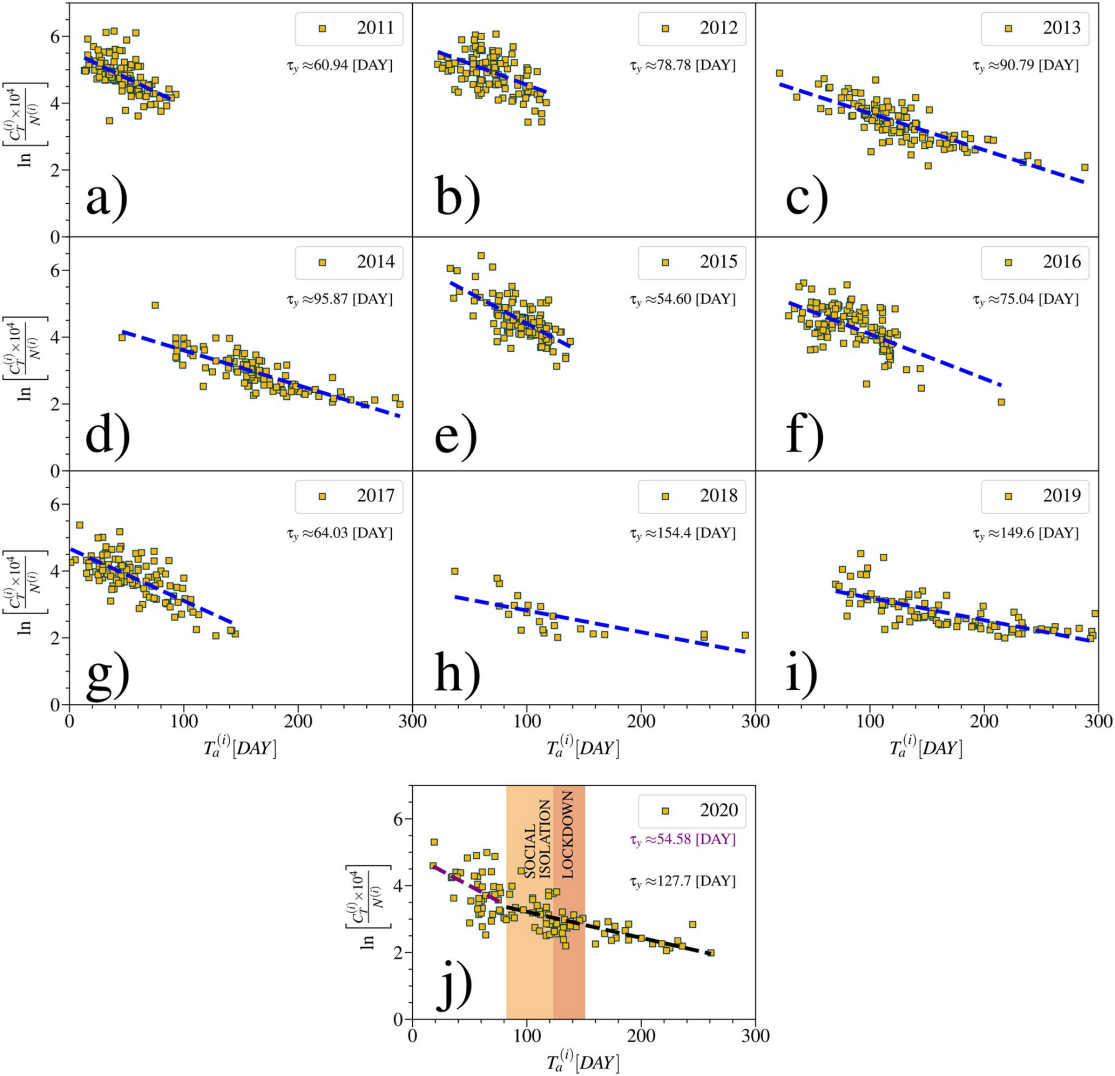
Exponential relation between total incidence of dengue cases, $C_{T}^{\left(i\right)}/N^{\left(i\right)},$ and the arrival time, $T_{a}^{i}$, of the dengue virus for each neighborhood in the city of Fortaleza. In (a)-(j) are the relations $C_{T}^{\left(i\right)}/N^{\left(i\right)}$ against $T_{a}^{i}$. The dashed lines represent fits through the Eq (2). Thus, it is possible to obtain the characteristic time of spread of the disease *τ*_*y*_ for each analyzed year. In the particular case of 2020, as shown in (j), due to the changes in urban mobility, a significantly different contamination pattern can be observed. It is possible to estimate a value of *τ*_*y*_ for before the implementation of mobility restrictions, $\tau_{2020}^{\left(T_{a}<\bar{T_{Lock}}\right)}\approx 54.6$ days (dashed purple line), and another for after the implementation of such restrictions, $\tau_{2020}^{\left(T_{a}>\bar{T_{Lock}}\right)}\approx 127.7$ days (dashed green line). Here, $\bar{T_{Lock}}=82$ days (March 22nd) is the effective time of enforcement of mobility restrictions.

The impact of mobility restrictions on inter-city dissemination of contagious diseases originating from an initial outbreak location (OL) has been investigated in Ref. [26], revealing that the arrival time of the disease *T*_*a*_ in a particular region is negatively correlated with the intensity of displacement from the OL to that region. Accordingly, the disease tends to arrive more quickly in regions with a higher degree of effective connectivity due to mobility. Thus, reductions in the number of rides between regions lead to an increase in *T*_*a*_ and, as a result, a decrease in the total number of cases due to the reduced speed of dissemination. In order to investigate if similar behavior occurs within a city, specifically at the neighborhood scale, we examined the correlation between the total incidence of dengue cases $C_{T}^{\left(i\right)}/N^{\left(i\right)}$ and the arrival time of the disease in each neighborhood, where $C_{T}^{\left(i\right)}$ and *N*^(*i*)^ refer to the total number of cases and local population in each neighborhood *i*, respectively. Fig 6a–6I show that indeed $C_{T}^{\left(i\right)}/N^{\left(i\right)}$ decays exponentially with $T_{a}^{\left(i\right)}$,

$$\begin{array}{c} \frac{C_{T}^{\left(i\right)}}{N^{\left(i\right)}}∼\text{exp}\;(\frac{-T_{a}^{\left(i\right)}}{\tau_{y}}), \end{array}$$ (2)

where *τ*_*y*_ denotes a characteristic time for year *y*. In each analyzed year, $T_{a}^{\left(i\right)}$ represents the number of days, starting from the first day of the year, necessary for a neighborhood *i* to effectively reach a specific total number of accumulated cases C* per 10,000 individuals. Here, we assume that C* = 7 is a reasonable number of cases to indicate the arrival of the disease in a given region. Variations in the value of C* do not significantly alter the exponential behavior of the curves plotted in Fig 6a–6I (see S4 Fig). Neighborhoods initially infected exhibit a higher number of cases. This pattern is observed in all years prior to 2020. Epidemic years are characterized by *τ*_*y*_ < 90 days, while non-epidemic years are well-represented by *τ*_*y*_ > 90 days (S5 Fig). In epidemic years, contamination takes place rapidly and escalates in neighborhoods until the conclusion of the first three months of the year. Non-epidemic years feature late contamination across different neighborhoods in the city. During these years, some outbreaks occur post-rainy season, with contamination spreading across all neighborhoods by July. The exponential decay observed in Eq 2 for the year 2020 is different from the previous ones. The Fig 6j shows zones of different regimes in the relationship $\text{ln}\;(C_{T}^{\left(i\right)}\times 10^{4}/N^{\left(i\right)})$ against $T_{a}^{\left(i\right)}$. Neighborhoods that were infected before the restriction period exhibit faster propagation compared to those infected later, implying in the reduction of the total number of cases. The disruption in the contagion rate, as seen in Fig 6j, is strong evidence that urban mobility plays a relevant role in the levels of dengue contamination within the city.

**Fig 7:**
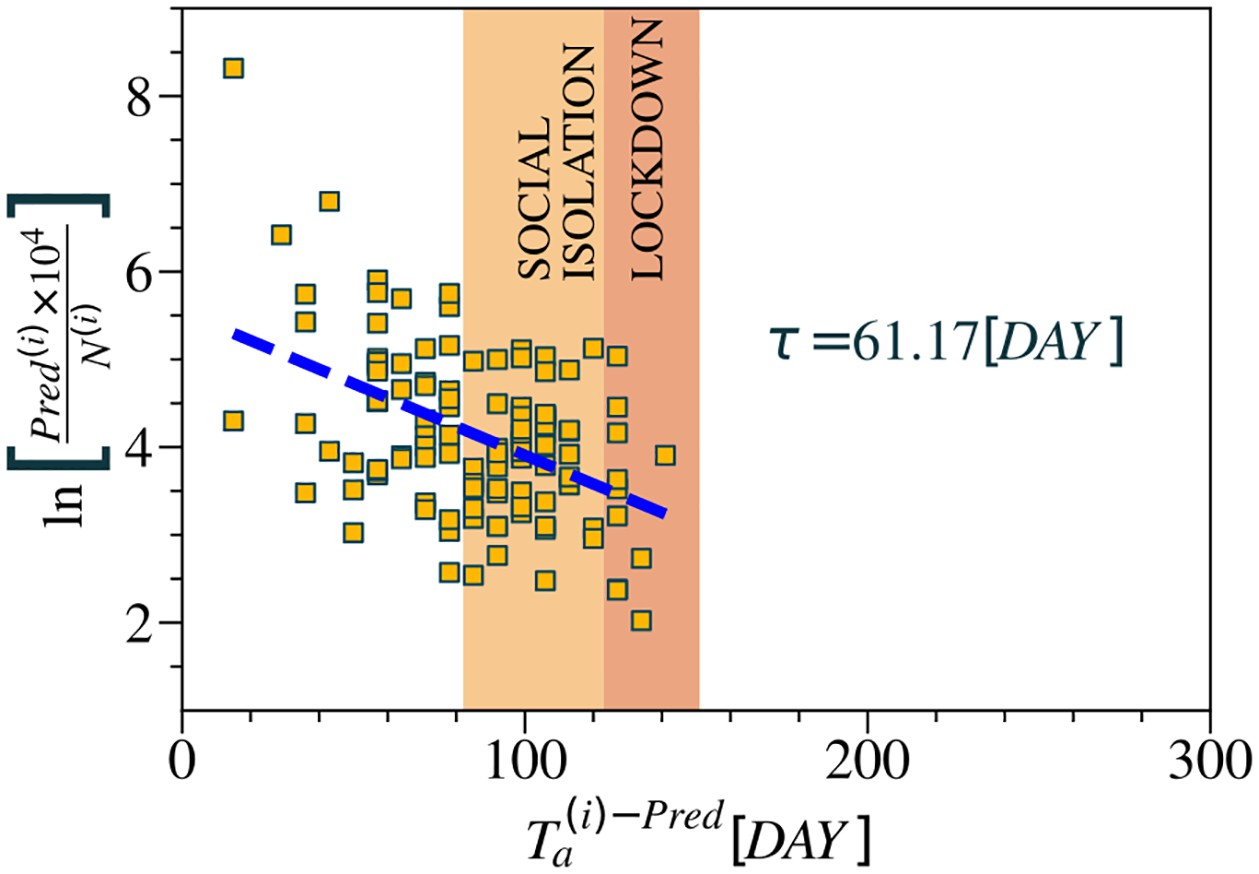
Prediction of the effective time of dengue transmission in 2020. Using a neural network model, it was possible to estimate the total number of dengue cases for each neighborhood *i* of the city of Fortaleza in 2020 in a hypothetical scenario where mobility restrictions had not been implemented. Such measure is observed at the end of the year and stored at the variable *Pred*^(*i*)^. We compute the time series that was predicted for each neighborhood during the year 2020 and calculate the time of arrival of the predicted disease from the network, $T_{a}^{(i)-Pred}$. The relation $Pred_{T}^{\left(i\right)}\times 10^{4}\;N^{\left(i\right)}$ against $T_{a}^{\left(Pred-i\right)}$ follows an exponential behavior, as expressed by Eq (2). Thus, it is possible to estimate the effective time of transmission of the disease where *τ* ≈ 61 days. Therefore, our predictions indicate that in 2020 dengue would contaminate neighborhoods in Fortaleza more quickly if there were no implementation of measures restricting urban mobility.

Finally, through the predictions made from the neural network, we obtained the total number of predicted cases for each neighborhood, *Pred*^(*i*)^, in a scenario where no restrictions on urban mobility were implemented. For late infected neighborhoods, we calculated the predicted disease arrival time $T_{a}^{\left(Pred-i\right)}$, considering C* = 7. As shown in Fig 7, the exponential relationship $Pred_{T}^{\left(i\right)}\times 10^{4}/N^{\left(i\right)}$ against $T_{a}^{\left(Pred-i\right)}$ highlighting the effective time *τ* = 61.17 days represents another possible evidence of the rapid spread of the disease in environments comparable to epidemic years in the absence of restrictions on urban mobility.

## Discussion

It is known that approximately 80% of breeding sites are found inside houses or in peridomiciliary environment, where female aedes lay their eggs in bottles, cans and small plastic containers found in backyards and vacant lots [27, 28]. Consequently, it is expected that a significant proportion of dengue infections should occur within the patient’s own residence. For local transmission to take place in an area previously unexposed to the disease, such as a specific neighborhood, both the presence of Aedes mosquitoes and an index case, often of external origin, are necessary. Nonetheless, the exact impact of the movement of infected individuals on the spread of dengue in a large urban setting remains ambiguous. Mobility restrictions can yield varied impacts based on the density and movement of the population. Specifically, isolating endemic regions may reduce dengue cases, whereas complete isolation might paradoxically increase them [19]. The incidental imposition of human mobility restrictions, including the extreme measure of lockdown, during the SARS-Cov-2 pandemic, inadvertently provided a unique opportunity to evaluate the effects of substantial mobility disruptions on the spread of other communicable diseases, including dengue. Although mosquito breeding sites within households and the peridomiciliary environment are critical in driving dengue transmission under normal mobility conditions, the restrictions on urban mobility during the COVID-19 pandemic likely disrupted the regular movement of individuals who serve as vectors between infected and non-infected areas. Consequently, while the household remains a key transmission site, the reduction in mobility between neighborhoods may have lessened the introduction of new infections from external sources, which would normally contribute to the spread of dengue across urban areas.

Our findings suggest that while the primary breeding grounds for Aedes aegypti remain inside homes and surrounding environments, the role of human mobility in transporting the virus between neighborhoods is essential for the broader transmission dynamics observed in urban settings. The lockdown measures, by limiting this movement, likely reduced the rate of new infections being carried from neighborhood to neighborhood. Despite some methodological limitations affecting the correlation with disease transmission, Aedes aegypti infestation indices, ascertained through entomological surveys, have consistently indicated widespread mosquito adaptation across Fortaleza [29]. This suggests that the movement of viremic individuals between different areas, such as from one neighborhood to another, could be a key factor in the initiation of an epidemic. This emphasizes the role of human mobility in not only connecting breeding sites but also spreading dengue across the city. The results of our study, showing a notable reduction in cases in neighborhoods that typically see higher transmission rates, align with this understanding. By limiting human movement, the opportunity for infected individuals to spread dengue beyond their immediate household is reduced, mitigating the overall transmission dynamics.

Our analysis proposes that reductions in urban mobility can significantly influence the onset of new disease outbreaks, particularly in neighborhoods initially affected by the disease. In essence, urban mobility restrictions appear to modify the dynamics of dengue transmission. In the initial outbreak locations, a notable discrepancy was observed between the total number of cases predicted using a neural network model and the actual observed data. As a result, neighborhoods infected at later stages experienced a reduced impact from the implementation of restrictive measures like lockdowns. These findings imply that mobility restrictions can effectively reduce the number of dengue cases, though their impact varies across different areas within a city. Such insights are essential for guiding governmental strategies to manage diseases with transmission dynamics similar to those of dengue.

Moreover, climatic factors, the distribution and types of mosquito breeding sites, and the level of immunity to circulating DENV serotypes may have also played a role in the observed decrease in dengue cases. However, the suspension of house-to-house vector control activities limits our ability to evaluate changes in Aedes aegypti infestation levels. Additionally, the potential increase in underreporting of dengue cases should be considered, as many primary health care units were forced to prioritize symptomatic respiratory patients over other services during the pandemic. Nonetheless, if underreporting did occur, it likely affected the reporting of mild cases more than severe cases, which generally require medical attention and hospitalization. Epidemiological data from Fortaleza suggest that the ratio of severe to mild dengue cases has remained consistent with pre-pandemic levels. Furthermore, our findings align with previous studies that have explored the relationship between mobility restrictions and dengue transmission in different regions. For instance, while Cavani *et al*. [16] suggest that lockdowns may increase transmission in residential environments, Liyanage *et al*. [17] found a correlation between reduced mobility and decreased dengue cases in Sri Lanka. These contrasting outcomes highlight the complexity of dengue transmission dynamics and underscore the need for localized approaches to managing mobility during epidemics. Our study adds to this body of work by providing evidence from an urban setting in Brazil that there is a strong correlation between mobility restrictions and the reduction in dengue cases.

## Conclusion

COVID-19 prompted various governmental actions concerning urban mobility in order to mitigate its proliferation. Generally, actions like *Lockdown* lead to significant reductions in the total number of cases due to decreased contact between individuals. However, the impact of these restrictive measures on other diseases with different transmission methods, especially in urban areas, remains unclear. Thus, using previously validated predictive models, we estimated the evolution of dengue cases in 2020 as if there had been no mobility restrictions and assessed the impacts of the actual reduction in urban mobility on dengue cases in each neighborhood of Fortaleza, Brazil.

The predictive method based on neural networks used in our study aligned with experts’ expectations before the mobility restrictions in 2020, anticipated an epidemic year for dengue cases. However, this expectation did not materialize, as dengue cases ceased to rise concurrently with the implementation of mobility restrictions. More precisely, these restrictive measures, initially implemented to mitigate COVID-19 transmission, have inadvertently demonstrated a remarkable ancillary benefit by contributing to a potential 72% reduction in observed dengue cases in Fortaleza in 2020, as predicted here using a neural network model. Additionally, our analysis using an ITS model corroborates these findings, indicating a reduction of approximately 45% in dengue cases due to mobility restrictions. Despite the numerical differences in the impact observed between the models, both models converge on the same conclusion, emphasizing the critical role of urban mobility restrictions in reducing the spread of diseases like dengue.

However, some limitations of our analysis should be considered. First, while the models provide strong evidence of the impact of mobility restrictions, they rely on available data, which may contain biases, such as underreporting of dengue cases during the COVID-19 pandemic. Moreover, the models do not account for changes in vector control efforts, which may have influenced the observed reduction in cases. It is also important to highlight that neither the LSTM nor the ITS models used mobility data directly; instead, they relied on historical transmission trends from years without mobility restrictions. As a result, the models capture the natural dynamics of dengue transmission under normal urban mobility conditions. Future studies should focus on integrating mobility data to enhance the accuracy of predictions.

## Supporting information

S1 Table Hyperparameters used to train the neural network. (PDF)

S1 FigAnnual distribution of dengue cases in Fortaleza (2011–2020).This figure comprises 10 individual maps, labeled from (a) to (j), which show the total number of dengue cases per neighborhood in Fortaleza for each year from 2011 to 2020. In each map, the variable $C_{T}^{\left(i\right)}$ represents the total number of cases observed in each neighborhood, visually expressed through a color gradient. The base map data was obtained from the public repository of the Brazilian Institute of Geography and Statistics (IBGE), accessible at https://geoftp.ibge.gov.br/organizacao_do_territorio/malhas_territoriais/malhas_de_setores_censitarios__divisoes_intramunicipais/censo_2010/setores_censitarios_shp/ce/ce_setores_censitarios.zip. These data are provided under a CC BY 4.0 license. Further information on the policies for access to geospatial data is available at the National Spatial Data Infrastructure (INDE): https://www.inde.gov.br/pdf/20@Decreto6666_27112008.pdf.(TIFF)

S2 Fig Correlogram of the total number of dengue cases for each 119 neighborhood in several years. We computed the Spearman correlation coefficient *ρ* between the pairs of years in the interval [2011, 2020]. The calculation of *ρ* involves comparing the ranking of the total number of dengue cases in each neighborhood for a pair of years analyzed. Thus, the correlation will be high when observations have a similar rank. Our analysis demonstrates that there is, in fact, a tendency to maintain the order of infection by neighborhoods. The distribution of correlation values, *g*(*ρ*), can be seen in the inset of the Figure, where 〈*ρ*〉 = 0.78 identifies the average of all pairs of years analyzed.
(TIFF)

S3 Fig Time series of the number of dengue cases (actual and predicted) for the city of Fortaleza from 2011 to 2020. The solid blue curves represent the actual time series data, while the red curves show the averages of the total cases from five predictions made for each year, along with the 90% confidence interval (C.I. 90%). The predictions were made using the LSTM model, following the training and validation protocol described in Table 1 of the main text.
(TIFF)

S4 FigDetermining the Threshold Value of C*.Figure shows the behavior of the Spearman correlation coefficient *ρ* between $\text{ln}\;(C_{T}^{\left(i\right)}\times 10^{4}/N^{\left(i\right)})$ and $T_{a}^{\left(i\right)}$ as a function of C*. Each box plot represents the distribution of correlation values for all the years analyzed from 2011 to 2020. We consider C* = 7 as the threshold number of accumulated cases per 10,000 inhabitants necessary for the disease to be effectively present in a neighborhood. It is observed that from C* = 7 onwards, the Spearman correlation tends to remain consistent.(TIFF)

S5 FigRelation between the effective time of dengue transmission and the total number of cases observed in the city of Fortaleza.From the Eq (2) in the main text it was possible to extract the value of *τ*_*y*_ for each endemic year. Here, $C_{T}^{\left(city\right)}=\sum_{i}C_{T}^{\left(i\right)}$ represents the total number of cases observed in the city in a certain year. Effectively, the longer the effective transmission time, the lower the number of cases observed in the city.(TIFF)
