# Supplementary figures and images for: The impact of COVID-19 mobility restrictions on dengue transmission in urban areas
Source: PLoS Negl Trop Dis. 2024 Nov 25;18(11):e0012644. doi: 10.1371/journal.pntd.0012644 (PMC11627415; doi:10.1371/journal.pntd.0012644)

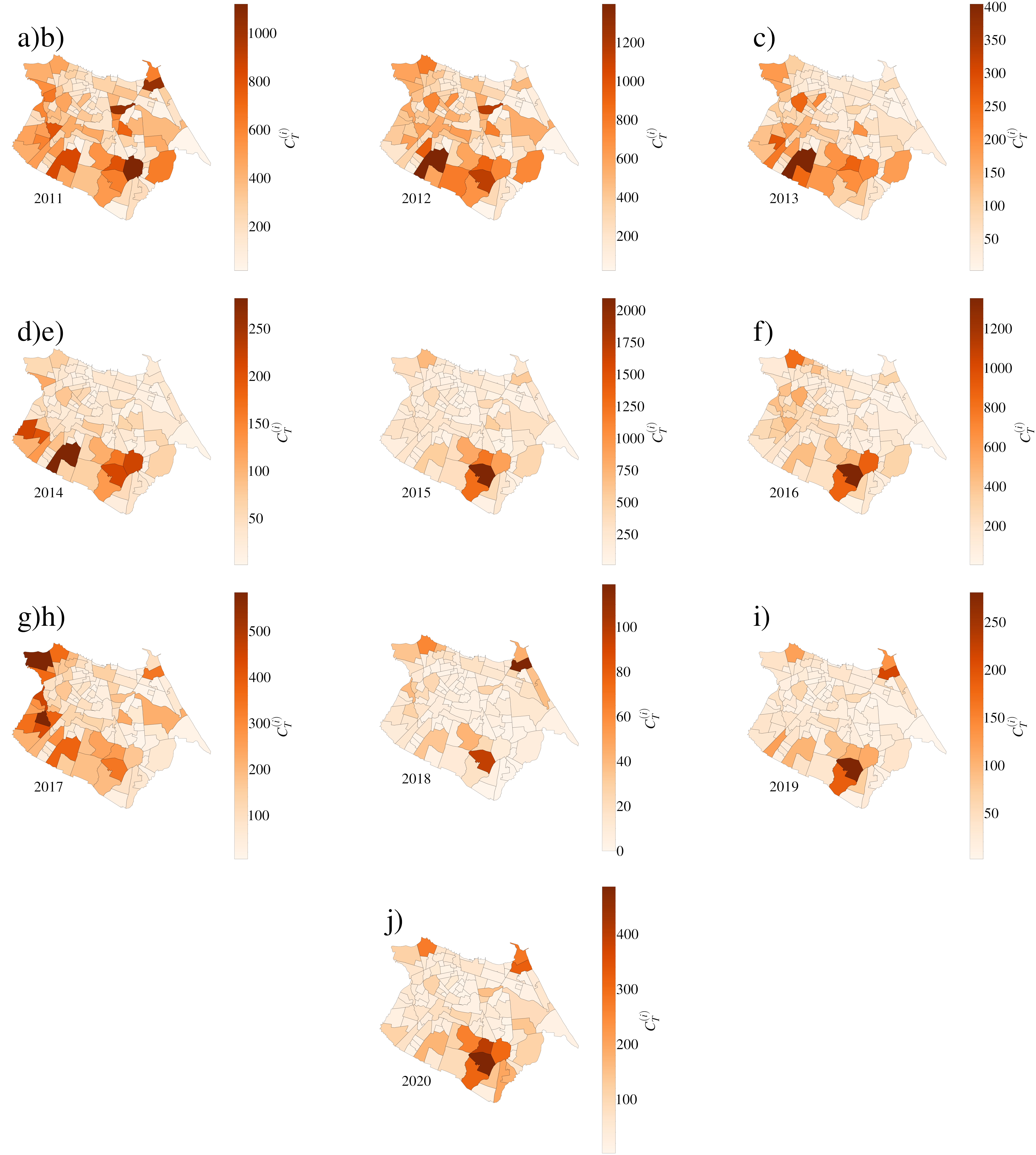

Supplement: S1 Fig — This figure comprises 10 individual maps, labeled from (a) to (j), which show the total number of dengue cases per neighborhood in Fortaleza for each year from 2011 to 2020. In each map, the variable CT(i) represents the total number of cases observed in each neighborhood, visually expressed through a color gradient. The base map data was obtained from the public repository of the Brazilian Institute of Geography and Statistics (IBGE), accessible at https://geoftp.ibge.gov.br/organizacao_do_territorio/malhas_territoriais/malhas_de_setores_censitarios__divisoes_intramunicipais/censo_2010/setores_censitarios_shp/ce/ce_setores_censitarios.zip. These data are provided under a CC BY 4.0 license. Further information on the policies for access to geospatial data is available at the National Spatial Data Infrastructure (INDE): https://www.inde.gov.br/pdf/20@Decreto6666_27112008.pdf. (TIFF) [file pntd.0012644.s002.tiff]

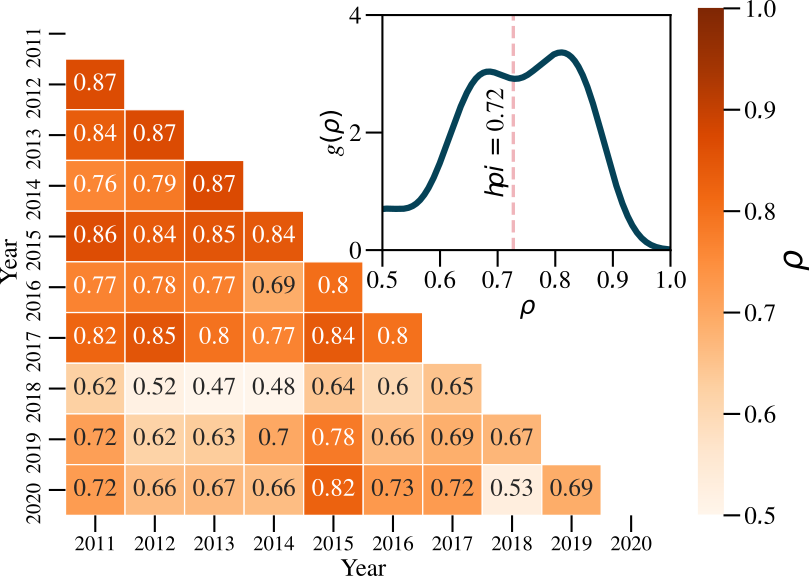

Supplement: S2 Fig — We computed the Spearman correlation coefficient ρ between the pairs of years in the interval [2011, 2020]. The calculation of ρ involves comparing the ranking of the total number of dengue cases in each neighborhood for a pair of years analyzed. Thus, the correlation will be high when observations have a similar rank. Our analysis demonstrates that there is, in fact, a tendency to maintain the order of infection by neighborhoods. The distribution of correlation values, g(ρ), can be seen in the inset of the Figure, where 〈ρ〉 = 0.78 identifies the average of all pairs of years analyzed. (TIFF) [file pntd.0012644.s003.tiff]

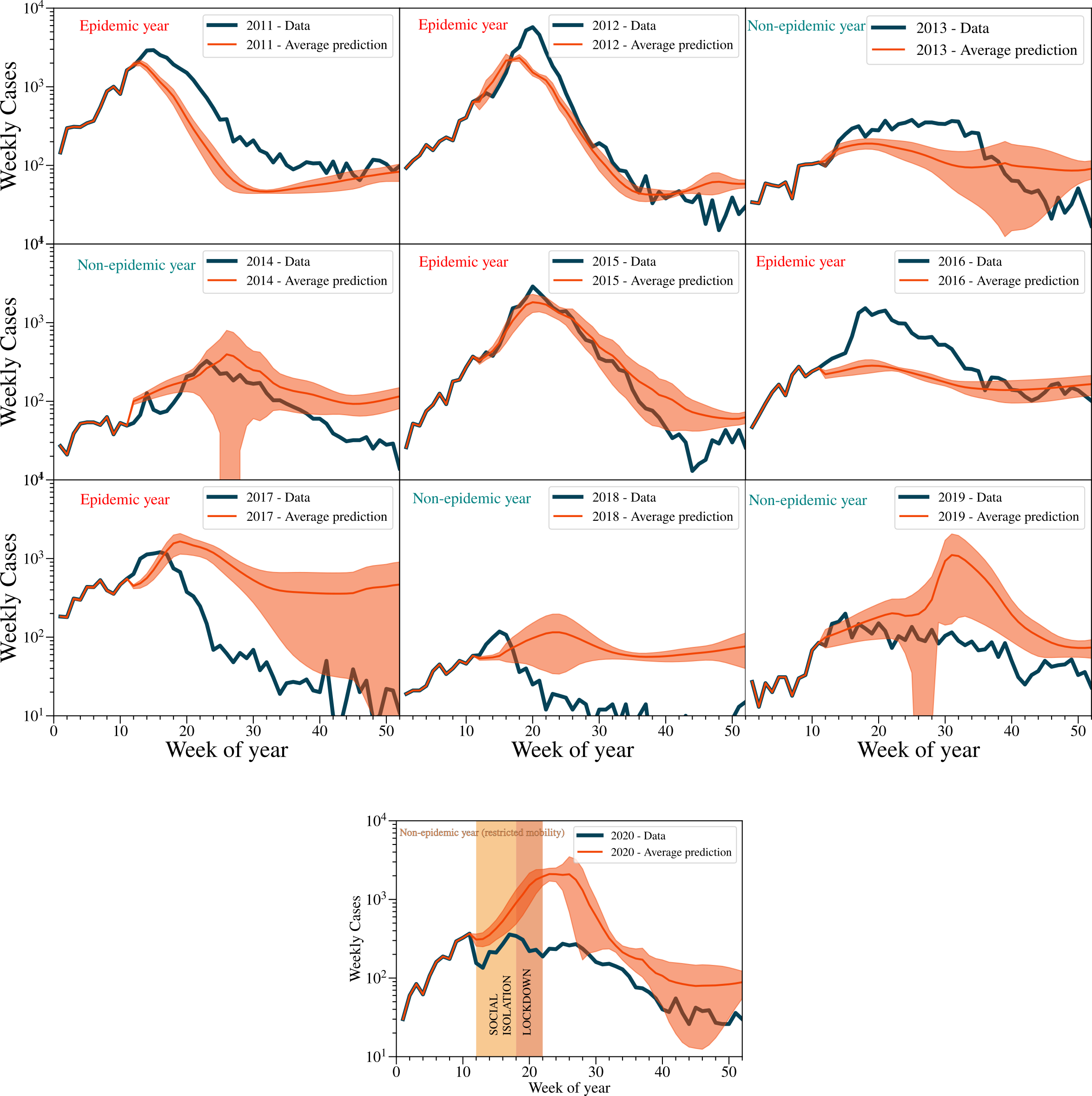

Supplement: S3 Fig — The solid blue curves represent the actual time series data, while the red curves show the averages of the total cases from five predictions made for each year, along with the 90% confidence interval (C.I. 90%). The predictions were made using the LSTM model, following the training and validation protocol described in Table 1 of the main text. (TIFF) [file pntd.0012644.s004.tiff]

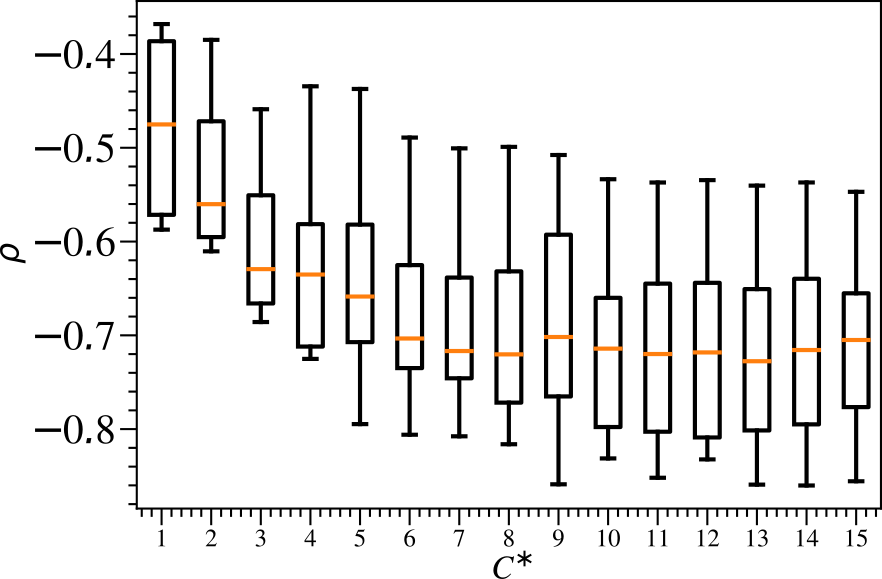

Supplement: S4 Fig — Figure shows the behavior of the Spearman correlation coefficient ρ between ln(CT(i)×104/N(i)) and Ta(i) as a function of C*. Each box plot represents the distribution of correlation values for all the years analyzed from 2011 to 2020. We consider C* = 7 as the threshold number of accumulated cases per 10,000 inhabitants necessary for the disease to be effectively present in a neighborhood. It is observed that from C* = 7 onwards, the Spearman correlation tends to remain consistent. (TIFF) [file pntd.0012644.s005.tiff]

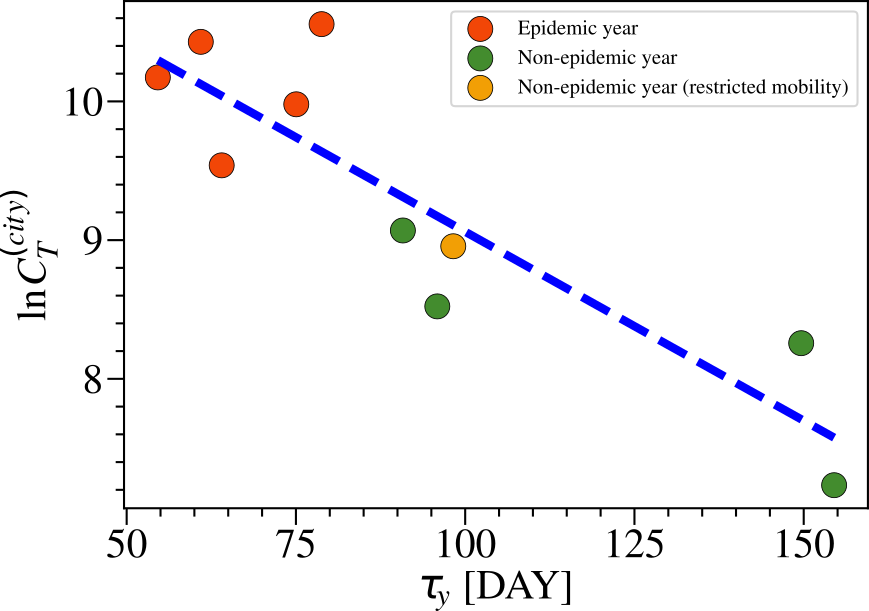

Supplement: S5 Fig — From the Eq (2) in the main text it was possible to extract the value of τy for each endemic year. Here, CT(city)=∑iCT(i) represents the total number of cases observed in the city in a certain year. Effectively, the longer the effective transmission time, the lower the number of cases observed in the city. (TIFF) [file pntd.0012644.s006.tiff]
